# Technical considerations in detection of HERV-K in amyotrophic lateral sclerosis: selection of controls and the perils of qPCR

**DOI:** 10.1186/s40478-019-0753-z

**Published:** 2019-07-03

**Authors:** Marta Garcia-Montojo, Wenxue Li, Avindra Nath

**Affiliations:** Section of Infections of the Nervous System, National Institute of Neurological Disorders and Stroke, National Institutes of Health, Bethesda, MD USA

**Keywords:** Amyotrophic lateral sclerosis, HERV-K, Human endogenous retroviruses

## Main text

The study by Garson et al. [12] failed to show a difference between the expression levels of Human endogenous retrovirus K (HERV-K) in amyotrophic lateral sclerosis (ALS) brain samples and controls by qPCR. However, several technical aspects need to be considered for the interpretation of their findings. Nearly half (11/23) the control samples had cancer. It is well known that HERV-K is activated in several types of cancer such as teratocarcinoma, germ cell tumors, melanoma, ovarian, and prostate cancer [4, 5, 14, 17, 23, 24, 34] and its expression is associated with various features of malignant cells [3, 25, 28, 30, 35]. Cancer cells can release viral-like particles containing viral products [3, 5, 23, 30]. These products would be expected to circulate within the brain vasculature and extracellular space hence can easily be detected in brain extracts. This is consistent with our observations where we were able to detect HERV-K transcripts in brain of patients with systemic cancer without any brain metastasis [6]. Garson et al., also found high levels of HERV-K transcripts in patients with cerebral infarcts. This is not surprising. Necrosis of the brain is likely to induce repair mechanisms that would include the presence of progenitor and stem cells. HERV-K and other endogenous retroviral elements are activated in these cells types and play an important role in cellular proliferation, similar to what is seen in cancer [11]. This raises two important questions: what the proper controls are to use in such studies and how does one reconcile the similarly opposite effects of HERV-K and other retroviral elements in cell proliferation and neurodegeneration. We have shown that HERV-K is specifically expressed in cortical neurons in a subpopulation of patients (~ 30%) with ALS [6, 21]. We did not see similar levels of activation in patients with other types of neurodegenerative disorders such as Alzheimer’s disease [21] and Parkinson’s disease [6]. Although other groups have shown the activation of other transposable elements in Alzheimer’s disease [13]. Similarly, increased circulating levels of antibodies directed against the HERV-K env have been found in serum and CSF of ALS patients [2].

Another important consideration in such studies is that ALS is a heterogeneous syndrome. Several genetic mutations have been associated with the disease, which suggest different pathophysiological pathways in these subtypes. Further, the clinical phenotypes may also vary. Some patients have predominant lower motor neuron involvement while others have predominant upper motor neuron involvement. A recent study classified ALS into three categories based on RNA seq analysis of the brain. One of these groups had activation of transposable elements including HERV-K that represented 20% of the samples [32]. Thus, a large enough sample size is necessary to capture these patients.

Garson et al. were meticulous in the design of their PCR assay and they followed the standard recommendations, checking for amplification specificity of the primers and calculating PCR efficiencies. However, if the goal of the study is to quantify RNA transcripts by qPCR, then the integrity of the samples measured by the RNA integrity number (RIN), is key [9]. RIN can have values ranging from 1 (totally degraded RNA) to 10 (intact RNA) [16]. While reference genes can control for small differences in RNA concentration and integrity [9], up to fourfold difference can be found in the relative expression of certain genes when comparing highly intact to degraded RNA [10] and negative and positive correlations between the RNA integrity and the relative expression have been seen depending on the target gene [27], or more accurately, depending on the differential resistance to degradation of the target and the reference gene. Studies have shown that the most important factor affecting expression levels of housekeeping genes, regardless of the diagnosis or brain tissue type, is the RIN number [22]. In human autopsy samples some degradation is to be expected. Factors such as post-mortem interval, pH of the brain, duration on the ventilator, duration of brain death etc., can affect the integrity of the RNA [31]. In the study by Garson et al., some RIN values were as low as 5, meaning 50% of the RNA was degraded. Most importantly, average RIN number was significantly different in ALS and controls. In contrast, in the study by Li et al. [21] only samples that had a RIN > 8 were used for the qPCR study and the experimental groups were matched for this critical variable.

As the authors did in this study, to control for small differences in RNA integrity and total amount of starting RNA, the use of a reference gene is strictly recommended. The selected reference gene should not be regulated or at least be minimally regulated and it must be sensitive to RNA degradation [10], logically in a similar manner to the target gene, because it is known that reference genes show different sensitivity to RNA degradation [10]. Garson et al. followed the international guidelines for qPCR, applying software (qBase+ and RefFinder) to determine the most stable reference genes among experimental groups. They selected one that was stable among ALS and control samples but, as they clearly state, the controls were more degraded (lower RIN number). Therefore, by choosing the housekeeping genes with the lowest variation among experimental groups, they selected the least sensitive to detect variations due to RNA integrity. So, the normalizations they performed and the attempts to show that the RIN number does not correlate with the normalized levels of HERV-K are not reliable, making it impossible to interpret the data.

Detection of transcripts that may be activated poses additional challenges in neurodegenerative diseases since the very cells of interest are the ones that are in various stages of degeneration or may be apoptotic. Analysis of expression by qPCR, due to its dependence on the appropriate selection of housekeeping genes, might not be ideal in these type of diseases. In a study by Rydbirk et al. [29], the authors tested a panel of reference genes to define the stability of the transcripts in neurodegenerative diseases and controls and then, validated them by analyzing the relative expression of a gene involved in neurodegeneration (GSK3B). GSK3B has been consistently implicated in neurodegeneration [19]. Its expression has been found altered in Alzheimer disease (AD) [15, 33] and Parkinson’s disease (PD) [1, 18]. Several functional studies have shown that the active enzyme encoded by GSK3B is elevated in these diseases [36] and is responsible for the phosphorylation of key proteins such as tau [8, 20] and alpha-synuclein [7], which leads to their pathological aggregation in the brain. GSK3B is a key player in the formation of beta-amyloid plaques [26]. Despite its clear association with neurodegeneration and the higher levels in AD and PD reported by several studies, Rydbirk et al. [29] could not find any difference in the relative expression of transcripts of GSK3B between AD or PD and controls when they used the most stable reference genes for normalization. They could only find higher expression of GSK3B in AD and PD when they used the most common reference genes (GAPDH and beta-actin) or the ones that they found as least stable.

A better method to use in these conditions might be RNAseq, where the quantification of the expression of a gene is “number of transcripts/million of total reads”. This method would be more adequate for neurodegenerative diseases since it controls for the possible decrease in global transcriptional activity in the disease. However, even this technique would not consider the differential susceptibility to degeneration of the various cell types in the cortex. Another method might be to perform laser capture microdissection of the neurons and then analyze the cells by RNA seq. The expression of the transcripts can also be visualized by in situ hybridization in tissue sections. Thus, due to the complexity of the problem and several technical challenges, no reliable conclusion can be made from the study by Garson et al., but further studies are needed using alternative methods and appropriate controls from brain samples with preserved integrity of the RNA to explore the expression of HERV-K in ALS.

## Abbreviations

AD: Alzheimer disease
ALS: Amyotrophic lateral sclerosis
HERV-K: Human endogenous retrovirus K
PD: Parkinson disease
RIN: RNA integrity number
